# Avian *Plasmodium* spp. and *Haemoproteus* spp. parasites in mosquitoes in Germany

**DOI:** 10.1186/s13071-023-05965-0

**Published:** 2023-10-18

**Authors:** Katharina Köchling, Günter A. Schaub, Doreen Werner, Helge Kampen

**Affiliations:** 1 Friedrich-Loeffler-Institut, Federal Research Institute for Animal Health, Greifswald, Germany; 2 Ruhr University Bochum, Bochum, Germany; 3 Leibniz Centre for Agricultural Landscape Research, Muencheberg, Germany

**Keywords:** Avian malaria, Culicidae, Haemosporida, Mosquito vectors

## Abstract

**Background:**

Although haemosporidian parasites may cause considerable health and economic problems in aviaries, there is limited understanding of the vectors transmitting them. Mosquito-borne *Plasmodium* species are responsible for the deaths of numerous exotic (= immunologically naïve) birds in zoos every year, while native birds are adapted to the parasites and largely protected by an effective immune response.

**Methods:**

Mosquitoes were collected in bird/animal parks, wetlands and private gardens in various regions of Germany from 2020 to 2022. Females were pooled with up to 10 specimens according to taxon, location and date. Extracted DNA was screened for avian Haemosporida-specific mitochondrial rDNA using real-time polymerase chain reaction (PCR). Positive samples were amplified by a *Plasmodium*/*Haemoproteus*-specific nested PCR targeting the partial cytochrome *b* gene, followed by sequencing of the PCR product for species identification. Sequences were checked against GenBank and MalAvi databases.

**Results:**

PCR of 2633 pools with 8834 female mosquitoes signalled infection with *Plasmodium* in 46 pools and with *Haemoproteus* in one pool. Further amplification and sequencing demonstrated the occurrence of *Haemoproteus majoris* lineage PARUS1 (*n* = 1) as well as several *Plasmodium* species and lineages, including *Plasmodium relictum* SGS1 (*n* = 16) and GRW11 (*n* = 1), *P. matutinum* LINN1 (*n* = 13), *P. vaughani* SYAT05 (*n* = 10), *P. circumflexum* TURDUS01 (*n* = 3), *P. cathemerium* PADOM02 (*n* = 1) and *Plasmodium* sp. SYBOR02 (*n* = 1) and PLOPRI01 (*n* = 1). The infections were detected in *Culex pipiens* sensu lato (*n* = 40), *Culiseta morsitans*/*fumipennis* (*n* = 6) and *Aedes cinereus*/*geminus* (*n* = 1).

**Conclusions:**

Although the overall *Plasmodium* minimum infection rate (5.2) appears to be low, the results demonstrated not only the ongoing circulation of *Plasmodium* parasites in the German mosquito population, but also the occurrence of eight distinct *Plasmodium* lineages, with three of them (PADOM02, SYBOR02, PLOPRI01) being detected in Germany for the first time. This study highlights the importance of conducting mosquito-borne pathogen surveillance studies simultaneously targeting vectors and vertebrate hosts, as certain species may be detected more readily in their vectors than in their vertebrate hosts, and vice versa.

**Graphical Abstract:**

**Figure:**
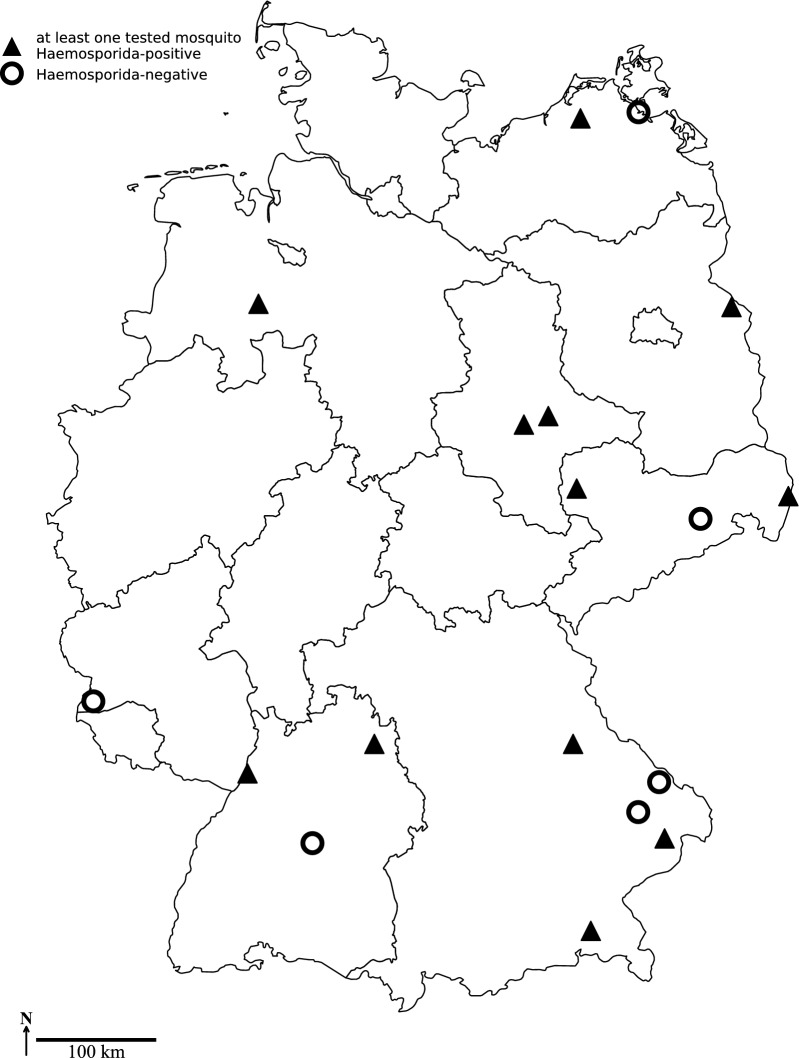

## Background

Avian malaria parasites (genus *Plasmodium*) are protozoan parasites with a global distribution, excluding the polar regions. Together with other genera, such as the closely related genera *Haemoproteus* and *Leucocytozoon*, they belong to the order Haemosporida [1]. The three genera share a similar and complex life cycle. Their member species are heteroxenous and sexually reproduce in haematophagous species of various nematoceran families while undergoing asexual reproduction in birds as intermediate hosts [1].

Species of the genus *Haemoproteus* are known to be transmitted by biting midges (Ceratopogonidae) and louse flies (Hippoboscidae). Infections of birds have commonly been considered harmless [2], but more recent data show reduced parental care in some species [3]. *Leucocytozoon* species are generally transmitted by black flies (Simuliidae), but some species are ceratopogonid-borne. Infection with *Leucocytozoon* spp. appears to be particularly severe in poultry [1]. For *Plasmodium* species, mosquitoes (Culicidae) are the main vectors [1]. Most native wild birds possess an efficient immune response due to coevolution with, and adaption to, syntopic plasmodial parasites, resulting in mild or no symptoms after infection [4]. However, exotic birds such as penguins, introduced from regions without Haemosporida, are highly susceptible to infection, especially with *Plasmodium* spp. [5], leading to numerous fatalities in zoos, bird parks and private aviaries every year [6].

The primary vectors of avian plasmodia are mosquito species belonging to the genus *Culex* [6], but species of the culicid genera *Aedes*, *Anopheles*, *Coquillettidia* and *Culiseta* are also known to be vector-competent for *Plasmodium* parasites [1, 2, 7, 8]. Despite being the most extensively studied genus within the order Haemosporida [8], the infection ecology and epidemiology of *Plasmodium* are not well understood, and prevalence data for potential vectors are scarce. In Germany, several studies have shown all three haemosporidian genera to occur in birds, with infection prevalence ranging from 1.9 to 31.3% for *Haemoproteus*, 9.5 to 85.3% for *Leucocytozoon* and 0.09 to 29.5% for *Plasmodium*, depending on bird species and location [9–12].

Until several decades ago, the identification of Haemosporida at the species level had been challenging. Only the introduction of advanced molecular techniques, including the collection of DNA sequences in databases, has facilitated quick detection and unambiguous taxonomic assignment. The shift from microscopy-based to molecular species identification also revealed a remarkable genetic diversity, resulting in the detection of different lineages, including several which appear to be distinct species [13].

To date, 55 morphologically distinct avian *Plasmodium* species have been identified globally [14], and a total of 1464 distinct lineages have been catalogued in the MalAvi database (http://130.235.244.92/Malavi/) established by Bensch et al. [13]. This public database collects avian haemosporidian data while focusing on the parasites’ mitochondrial cytochrome *b* (*cytb*) gene.

Apparently, the most common *Plasmodium* lineages in Europe, both in birds and mosquitoes, are *P. relictum* SGS1, *P. vaughani* SYAT05 and *P. matutinum* LINN1 [15–19]. These lineages have also been detected in birds in Germany [12, 20, 21], but not in mosquitoes. Only one study has demonstrated the presence of haemosporidian parasites in mosquitoes in Germany [22]. All three genera were found but were not identified to species level.

The present study follows on the study by Heym et al. [22] and aims to provide data on (i) the spatio-temporal haemosporidian infection prevalence in mosquitoes collected in Germany and (ii) the identification of parasite lineage diversity within different mosquito species. As avian *Plasmodium* parasites cause the most significant harm to captive exotic bird populations, the study focused on mosquitoes from bird and animal parks.

## Methods

### Mosquito collection and morphological identification

**Fig. 1:**
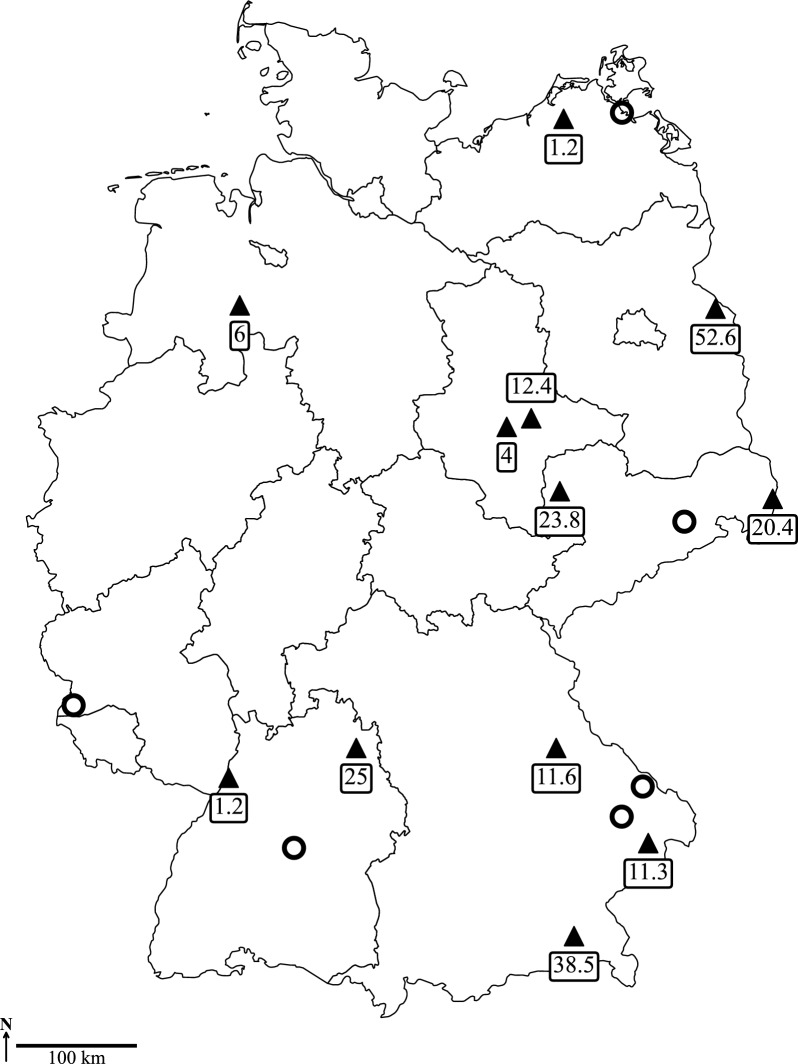
Sampling locations in Germany. Triangles: Locations with at least one mosquito pool found positive either with *Haemoproteus* or *Plasmodium*. Circles: Locations where all tested mosquito pools were negative. Figures in boxes represent local minimum infection rates averaged over all mosquito taxa and all collection years

Mosquito collection was carried out from April to October (occasionally in November) 2020 to 2021 and from April to September 2022. Three different kinds of biotopes were sampled using different trapping methods. BG-Sentinel traps (Biogents, Regensburg, Germany) equipped with BG-Lure (Biogents) and CO_2_ from a gas tank as attractants were activated weekly for 24 h by local trap attendants in bird/animal parks and in a peatland known for its high abundance of birds. In addition, mosquitoes were collected in a cellar (hibernation shelter) by an aspirator in one animal park in November 2020 and 2021, respectively. Encephalitis vector surveillance (EVS) traps (BioQuip, Compton, CA, USA) equipped with dry ice as a CO_2_ source were used to capture mosquitoes in floodplains along the Elbe River in the federal state of Saxony-Anhalt. Ten EVS traps were operated overnight once per month from July to September of each collection season. Additionally, popup garden bags, exposed in private gardens as resting sites for mosquito females during blood digestion [23], were sampled irregularly with an aspirator. In total, 18 locations were sampled, with a focus on eastern Germany (Fig. 1).

The mosquitoes were placed in a freezer (−20 °C or −80 °C) or on dry ice immediately after collection. In the laboratory, they were morphologically identified to species or complex/group level on a chilling table under a stereomicroscope, using the identification key of Becker et al. [24].

### Genetic processing of mosquitoes

For further examination, mosquitoes were generally pooled with up to 10 specimens according to species, trapping location and collection date. Due to their large size, *Culiseta* species were pooled with up to five specimens. All *Culex pipiens* complex pools found positive for Haemosporida DNA were retrospectively tested for species and biotype by real-time polymerase chain reaction (PCR) [25]. Species of the *An. maculipennis* complex and damaged specimens, for which morphological species identification was not possible, were processed individually using identification approaches described by Heym et al. [22]. Blood-fed mosquitoes were also processed individually.

DNA and RNA were simultaneously extracted from both pooled and single mosquitoes. Briefly, whole mosquitoes were homogenised by a TissueLyser II (Qiagen, Hilden, Germany) with three 3-mm steel beads (TIS, Gauting, Germany) in 500 µl (single mosquitoes) or 750 µl (pooled mosquitoes) of serum-free minimal essential medium (FLI-intern cell culture medium = Eagle’s minimum essential medium with Earle’s and Hank’s salts plus non-essential amino acids) [26]. To avoid microbial contamination, 5 µl (for single mosquitoes) and 7.5 µl (for mosquito pools) of a ready-to-use penicillin–streptomycin mixture and 1 µl (for single mosquitoes) or 1.5 µl (for mosquito pools) of a ready-to-use gentamicin–amphotericin mixture (Thermo Fisher Scientific, Dreieich, Germany) were added to the medium. The NucleoMag VET Kit (Macherey–Nagel, Düren, Germany) was used for nucleic acid extraction from 200 µl homogenate as described in the manufacturer’s protocol.

Samples were then screened for *Haemoproteus*, *Leucocytozoon* and *Plasmodium* species by amplifying a 182-base-pair (bp) fragment of the conserved mitochondrial ribosomal DNA (rDNA) region [27]. The screening was performed following the protocol described by Heym et al. [22]. Each real-time PCR included both a negative (nuclease-free water) and a positive control (synthetic *P. relictum* DNA, GenBank accession no. NC012426) and was run on a CFX96 Real-Time System (Bio-Rad, Munich, Germany). Data were analysed by high resolution melting-curve analysis using Bio-Rad CFX-Manager software.

For species and lineage identification within the genera *Haemoproteus* and *Plasmodium*, positive sample DNA was further amplified by a nested PCR, targeting a 477-bp fragment of the mitochondrial *cytb* gene [27]. Both of the nested PCRs were conducted with the Blue Probe qPCR Kit (Biozym Scientific, Hessisch Oldendorf, Germany), with 0.5 µM forward (H332F 1st PCR, H350F 2nd PCR) and reverse (HAEMNR2 1st PCR, HAEMR2 2nd PCR) primers (Table 1). Nuclease-free water was added to reach a total reaction volume of 20 µl.

**Table 1:** Primer sequences of the three PCRs used to detect *Plasmodium* and *Haemoproteus* parasites

Primer	Sequence	Amplicon length [bp]	Target region	Purpose of PCR	References
R330F	5′-CGTTCTTAACCCAGCTCACG-3′	182	mitochondrial rDNA	Screening (real-time PCR)	[27]
R480RL	5′-GCCTGGAGGTWAYGTCC-3′				
H332F	5′-GAGAATTATGGAGYGGATGGTG-3′	526	*cytb* gene	Identification (1st nested PCR)	[27]
HAEMNR2	5′-AGAGGTGTAGCATATCTATCTAC-3′				[42]
H350F	5′-GGTGTTTTAGATATATGCATGC-3′	477	*cytb* gene	Identification (2nd nested PCR)	[27]
HAEMR2	5′-GCATTATCTGGATGTGATAATGGT-3′				[43]

For the first nested PCR, 5 µl DNA templates were used, while 3 µl PCR products of the first nested PCR were transferred to the second nested PCR. The cycling started with an activation step for 2 min at 95 °C, followed by 20 cycles in the first nested PCR and 35 cycles in the second nested PCR of 30 s at 95 °C, 30 s at 60 °C, and 30 s at 72 °C, and a final elongation step for 5 min at 72 °C. The products of the second nested PCR were visualised on a 1.5% agarose gel by electrophoresis, excised, and extracted using the QIAquick Gel Extraction Kit (Qiagen). Extracts were sequenced bidirectionally with H350F and HAEMR2 primers using the Mix2Seq Kit (Eurofins Genomics, Ebersberg, Germany). Sequences were edited and aligned using the Geneious Prime programme, version 2021.0.1 (Biomatters, Auckland, New Zealand). The aligned sequences were compared with sequences from the MalAvi (http://130.235.244.92/Malavi/) and GenBank (www.ncbi.nlm.nih.gov) databases.

In addition, the blood source of engorged Haemosporida-positive mosquito females was determined. As the published detection systems have different sensitivities for different vertebrate groups, two PCR protocols were used: First, a conserved 16S rDNA region of vertebrates was amplified and sequenced as described by Kitano et al. [28]. If this DNA amplification failed, a second PCR was conducted with vertebrate-specific primers targeting the cytochrome *c* oxidase subunit 1 (*COI*) gene [29]. This PCR made use of the QuantiTect Multiplex qPCR NoROX Kit (Qiagen) and was carried out with 2.5 µl DNA template and primers at a concentration of 0.2 µM each, in a total reaction volume of 12.5 µl. The cycling conditions consisted of an initial activation step at 95 °C for 15 min, followed by 40 cycles of 95 °C for 60 s, 60 °C for 40 s and 72 °C for 60 s, and a final elongation step at 72 °C for 10 min. Sequencing was done as described, and the obtained sequences were compared with sequences deposited in GenBank (www.ncbi.nlm.nih.gov), with only sequence identities of at least 97% being accepted.

### Determination of minimum infection rate

In order to compare and evaluate infection prevalence, minimum infection rates (MIR) were calculated as described by the CDC [30]. The MIR assumes that a pool tested positive contains a single positive specimen, independent of the size of the pool.

## Results

### Mosquito species and detection of Haemosporida

In this study, 8834 females belonging to 25 species/species complexes were analysed. The majority of the analysed specimens were collected in the months of July and August and belonged to the *Cx. pipiens* complex (*n* = 3266; 37%), followed by *Aedes vexans* (*n* = 2145; 24.3%), *Aedes cinereus/geminus* (*n* = 1161; 13.1%), *Anopheles daciae* (*n* = 643; 7.3%), *Culex modestus* (*n* = 433; 4.9%), *Aedes sticticus* (*n* = 280; 3.2%), *Aedes annulipes* group (*n* = 255; 2.9%), *Culiseta morsitans/fumipennis* (*n* = 175; 2%), *Anopheles messeae* (*n* = 103; 1.2%), *Culiseta annulata* (*n* = 85; 1%), *Anopheles plumbeus* (*n* = 36; 0.4%), *Aedes communis* (*n* = 34; 0.4%), *Culex territans* (*n* = 33; 0.4%), *Aedes caspius* (*n* = 30; 0.3%), *Culiseta ochroptera* (*n* = 30; 0.3%), *Aedes cataphylla* (*n* = 19; 0.2%), *Aedes punctor* (*n* = 19; 0.2%), *Aedes detritus* (*n* = 17; 0.2%), *Aedes japonicus* (*n* = 16; 0.2%), *Anopheles claviger* (*n* = 16; 0.2%), *Coquillettidia richiardii* (*n* = 15; 0.2%), *Anopheles maculipennis* (*n* = 14; 0.2%), *Culex hortensis* (*n* = 7; 0.1%), *Aedes geniculatus* (*n* = 1; 0.01%) and *Aedes leucomelas* (*n* = 1; 0.01%).

A total of 2633 pools were created to be tested for avian Haemosporida, among which 46 mosquito pools (1.7%) contained DNA of *Plasmodium* and one pool (0.04%) DNA of *Haemoproteus* (Table 2). The *Plasmodium*-positive pools consisted of *Ae. cinereus* or *Ae*. *geminus* or a mixture of both (*Ae. cinereus*/*geminus*), *Cs. morsitans* or *Cs. fumipennis* or a mixture of both (*Cs. morsitans/fumipennis*), and taxa of the *Cx. pipiens* complex. *Haemoproteus* DNA was only found in *Cs. morsitans/fumipennis*.

**Table 2:** Total *Plasmodium* and *Haemoproteus* prevalence and minimum infection rates (MIR) per year and species

Year	Species	Pools tested [*n*]	Individuals tested [*n*]	*Plasmodium-*positive pools [*n*]	*Haemoproteus-*positive pools [*n*]	Positive pools [%]	MIR
2020	*Ae. cinereus*/*geminus*	22	84	0	0	0	na
	*Cs. morsitans*/*fumipennis*	1	1	0	0	0	na
	*Cx. pipiens* s.l.	319	1476	12	0	3.8	8.1
	Others	972	1494	0	0	0	na
	Total	1314	3055	12	0	0.9	3.9
2021	*Ae. cinereus/geminus*	151	1077	1	0	0.7	0.9
	*Cs. morsitans/fumipennis*	42	167	5	0	11.9	29.9
	*Cx. pipiens* s.l.	439	1485	23	0	5.2	15.5
	Others	432	2311	0	0	0	na
	Total	1064	5040	29	0	2.7	5.8
2022	*Cs. morsitans/fumipennis*	7	7	0	1	14.3	142.9
	*Cx. pipiens* s.l.	109	305	5	0	4.6	16.4
	Others	139	427	0	0	0	na
	Total	255	739	5	1	2.4	8.1

*na* not applicable

### *Plasmodium* and *Haemoproteus* prevalence and lineage diversity

The 47 positive pools represent nine distinct haemosporidian mitochondrial *cytb* lineages (Table 3). The most frequently detected one was *P. relictum* SGS1 (*n* = 16; 34%), followed by *P. matutinum* LINN1 (*n* = 13; 27.7%), *P. vaughani* SYAT05 (*n* = 10; 21.3%), *P. circumflexum* TURDUS1 (*n* = 3; 6.4%), *P. cathemerium* PADOM02 (*n* = 1; 0.5%), *P. relictum* GRW11 (*n* = 1; 0.5%), *Plasmodium* sp. SYBOR02 (*n* = 1; 0.5%), and *Plasmodium* sp. PLOPRI01 (*n* = 1; 0.5%). The single *Haemoproteus*-positive pool contained *Haemoproteus majoris* PARUS1 (0.5%). The *Plasmodium* sequences clearly matched with entries in GenBank but two pools of *P. relictum*, one pool of *P. matunitum* and one pool of *P. vaughani* matched less clearly with entries in the MalAvi database due to the presence of more than one lineage with similar sequence identities (Table 3). For the purpose of this study, we followed GenBank, but show findings with ambiguous MalAvi results in Table 3.

**Table 3:** Haemosporida species and lineages found

Haemosporida taxon	Lineage (MalAvi)	Accession number (GenBank)	Number of detections	Match (MalAvi) [%]	Length of PCR product^a^ [bp]	Mosquito taxa in positive pools	Pool size^b^	LP^c^ [%]	References
*P. cathemerium*	PADOM02	MK018109	1	100	477	*Cx. pipiens* biotype *pipiens*	1	0.5	[44]
*P. circumflexum*	TURDUS1	MF928791	3	100	477	*Cs. morsitans*/*fumipennis*	1	6.4	–
*P. matutinum*	LINN1	MK652234	12 (1^d^)	99–100	430–477	*Cx. pipiens* biotype *pipiens*	1	27.7	[45]
						*Cx. pipiens* biotype *pipiens*/*Cx. torrentium*	3		
*P. relictum*	SGS1	MK652232	14 (2^d^)	100	444–477	*Ae. cinereus*/*geminus*	1	34.0	[45]
						*Cx. pipiens* biotype *pipiens*	1		
						*Cx. pipiens* biotype *pipiens*/*molestus*	10		
*P. relictum*	GRW11	KY653772	1	100	477	*Cx. pipiens* biotype *pipiens*	1	0.5	[46]
*P. vaughani*	SYAT05	MK652242	9 (1^d^)	99–100	415–477	*Cx. pipiens* biotype *pipiens*	1	21.3	[45]
						*Cx. torrentium*	1		
						*Cs. morsitans*/*fumipennis*	1		
						*Cx. pipiens* biotype *pipiens*/*Cx. torrentium*	10		
*Plasmodium* sp.	SYBOR02	OP358412	1	100	477	*Cs. morsitans*/*fumipennis*	5	0.5	[47]
*Plasmodium* sp.	PLOPRI01	MG018694	1	100	477	*Cx. pipiens* biotype *pipiens*	1	0.5	[48]
*H. majoris*	PARUS1	KY451714	1	100	477	*Cs. morsitans*/*fumipennis*	1	0.5	[49]

^a^Nested PCR, targeting a 477 bp-fragment of the mitochondrial *cytb* gene [27]

^b^Number of mosquito specimens

^c^Lineage prevalence

^d^Samples showing clear sequence matching in GenBank but inconclusive results in the MalAvi database due to the presence of more than one lineage with similar sequence identities

In 2020, 12 out of 1314 pools (0.9%) contained *Plasmodium* DNA (Table 2), with all of them consisting of *Cx. pipiens* complex mosquitoes. Mosquitoes in 10 of those pools were identified as *Cx. pipiens* biotype *pipiens*, one pool was a mixture of *Cx. pipiens* biotype *pipiens* and *Cx. torrentium*, and the 12th positive pool either was a mixture of *Cx. pipiens* biotype *pipiens* and biotype *molestus* or contained hybrids of the biotypes. The most frequently detected *Plasmodium* species in 2020 was *P. matutinum* LINN1 (*n* = 5), followed by *P. relictum* SGS1 (*n* = 4) and *P. vaughani* SYAT05 (*n* = 3).

In 2021, 29 out of 1064 mosquito pools (2.7%) contained DNA of *Plasmodium*. Of those, 20 pools consisted of *Cx. pipiens* biotype *pipiens* and one pool of *Cx. torrentium*; one pool was a mixture of *Cx. pipiens* biotype *pipiens* and *Cx. torrentium*, and one pool was either a mixture of *Cx. pipiens* biotype *pipiens* and biotype *molestus* or a pool of hybrids of these biotypes. Five pools contained *Cs. morsitans/fumipennis* and one pool *Ae. cinereus/geminus.* More than 50% of the positive pools contained *P. relictum* SGS1 DNA (*n* = 11). *Plasmodium matutinum* LINN1 (*n* = 7) was the next most frequently found *Plasmodium*, followed by *P. vaughani* SYAT05 (*n* = 6) and *P. circumflexum* TURDUS1 (*n* = 3). In addition, one pool was found positive for *P. cathemerium* PADOM02 and one for *Plasmodium* sp*.* SYBOR02.

In 2022, six out of 255 pools (2.4%) contained DNA of Haemosporida. In five pools (2%), PCR demonstrated infections with *Plasmodium*, all of them containing only *Cx. pipiens* biotype *pipiens*. One pool (0.4%) consisting of *Cs. morsitans*/*fumipennis* was positive for *H. majoris* PARUS1. The other single positive reactions were assigned to *P. relictum* SGS1, *P. relictum* GRW11, *P. matutinum* LINN1, *P. vaughani* SYAT05 and *Plasmodium* sp. PLOPRI01.

### Minimum infection rates according to mosquito taxa and collection time

Over all years of sampling, the MIR was 5.3 (*Plasmodium*: 5.2; *Haemoproteus*: 0.1), but varied tremendously according to mosquito taxon, year and sampling location. Of the 18 locations surveyed for the presence of avian Haemosporida in mosquitoes, 12 turned out to be positive for at least one of the parasites (Fig. 1). The MIRs of the sampling locations ranged from 1.2 to 52.5 (Fig. 1).

**Fig. 2:**
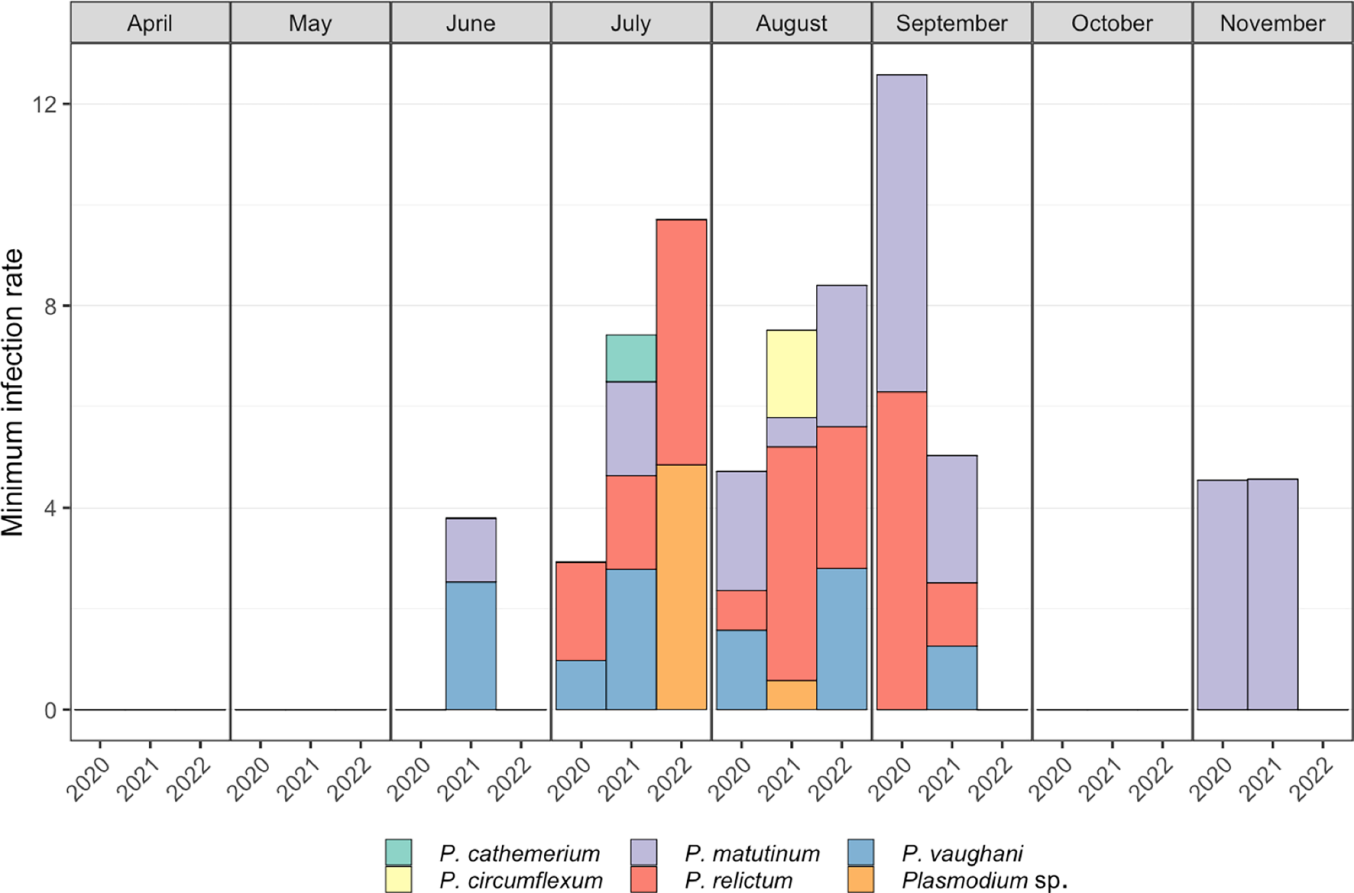
Minimum infection rates according to month and year. Different colours indicate minimum infection rates of different *Plasmodium* species

The highest MIRs were recorded for *Cs. morsitans*/*fumipennis* in 2022 (142.9 for *Haemoproteus*) and in 2021 (29.9 for *Plasmodium*) (Table 2). For specimens of the *Cx. pipiens* complex, the MIR varied between 8.1 and 16.4 across years. Although a large number of individuals were examined, only one pool of *Ae. cinereus*/*geminus* tested positive in the study in 2021, resulting in a very low MIR of 0.9 (Table 2). The MIRs for *Plasmodium* parasites were highest in September in 2020, and in July and August in 2021 and 2022 (Fig. 2). Finally, the MIRs were similar in November 2020 and 2021 (4.5 and 4.6, respectively) in mosquitoes collected in a hibernation shelter (cellar), where only *P. matutinum* was detected (Fig. 2).

### Mosquito blood meal analysis

Four mosquitoes (three *Cx. pipiens* biotype *pipiens* and one *Cs. morsitans*/*fumipennis* specimen) containing DNA of *Plasmodium* or *Haemoproteus* were engorged. The origin of the blood meals, however, could only be identified for two *Cx. pipiens* biotype *pipiens* specimens collected in different bird parks. According to GenBank, the blood host DNA of one mosquito containing DNA of *P. matutinum* LINN1 matched best to an African sacred ibis (*Threskiornis aethiopicus*). However, instead of this avian species only the closely related black-headed ibis (*Threskiornis melanocephalus*) was present in the sampled bird park (J. Westenfelder, pers. comm.). In turn, a *COI* DNA sequence of that species was not available from GenBank. *COI* DNA sequence analysis of the second mosquito which contained DNA of *P. relictum* SGS1 produced the common eider (*Somateria mollissima*) as a blood host which did belong to the bird population of the second bird park (G. Haase, pers. comm.).

## Discussion

Avian malaria is a huge problem in birdkeeping facilities, and the risk of infection by the causative parasites is increasing due to climate warming, with a particularly strong effect expected for the future in Europe and Africa [31]. Assessment of spatio-temporal natural circulation, identification of transmission hotspots and potential vectors, and estimation of infection prevalence are therefore basic steps in the management of the disease.

To our knowledge, this is the first report on the molecular detection and characterisation of avian *Plasmodium* and *Haemoproteus* lineages in mosquitoes from Germany. The present study assesses the prevalence of haemosporidian parasites in mosquitoes collected from 18 different locations across Germany from 2020 to 2022. A total of 25 mosquito species/species groups were included, with the taxa of the *Cx. pipiens* complex being the most frequently analysed and most frequently detected positive for *Plasmodium* infection.

The total mosquito infection prevalence for *Plasmodium* of 1.7% obtained in the present study exceeded that of 0.03% found in Portugal [15], but is relatively low in comparison to prevalences reported from various other European countries, with 6.4% in Austria [18], up to 15.8% in France [32], 15.7 to 19.5% in Spain [33] and 20.3% in Switzerland [16]. However, methodological approaches varied. While in Switzerland and Spain only one location was sampled, in France four locations close to each other, in Portugal four areas far apart and in Austria multiple sites across different provinces were trapped, similar to the present study. The mosquito infection prevalence seemed to be higher the smaller the sample areas were. In general, prevalence varied according to vector species, location and collection month [15, 16, 18, 32, 33] which is in agreement with our study.

However, in the context of analysing pooled samples, it is crucial to recognise the potential for underestimating the actual infection prevalence, as pointed out by Schoener et al. [18]. This became also evident in the present study, in which the method used was less sensitive in pooled than in individual mosquitoes (unpublished data). Despite this limitation, pooling mosquitoes was selected in favour of examining a large number of mosquitoes instead of the time-consuming and cost-intensive analysis of individual mosquitoes.

The analysis of the mosquito samples collected from various locations across Germany revealed that at 61.1% of the locations, at least one pool contained DNA of *Plasmodium.* This finding suggests a widespread distribution of avian *Plasmodium* parasites within the German mosquito population, which is consistent with studies on plasmodial infections in birds carried out in Germany [9–12]. The results of the present study, however, are of limited value regarding the determination of geographical infection prevalence. This is due to the fact that at certain locations examined in this study, the mosquito sample was relatively small.

Most studies on haemosporidians in mosquitoes focus primarily on the genus *Culex*, as species of this genus are known to play a significant role in transmitting avian *Plasmodium* parasites. Absolute numbers of positive mosquito pools in the present study appear to confirm a predominant role of the genus *Culex*. By contrast, when applying the MIR concept, *Cs. morsitans*/*fumipennis* exhibited the highest infection prevalence. The prevalence and MIR estimates should be interpreted with caution, as a small sample size may result in an artificially high prevalence or MIR due to an incidental finding. However, this is unlikely to be the case with *Cs. morsitans*/*fumipennis*, as several pools of a moderate sample size of these mosquitoes contained DNA of *Plasmodium* in 2021. The reverse might also apply: a single signal in a large sample resulting in a low MIR is likely to represent reality, as seen in the case of *Ae. cinereus*/*geminus* (Table 2).

As opposed to the taxa of the *Cx. pipiens* complex, which can be found throughout Germany in a variety of habitats, including artificial breeding sites, *Cs. morsitans*/*fumipennis* are generally less abundant and occur primarily in wet forests and meadows [24]. As a result, *Cx. pipiens* complex taxa are considered more important vectors of *Plasmodium* species. However, *Cs. morsitans*/*fumipennis* are known to have a preference for birds as hosts and can frequently be encountered in areas with high avian populations, such as the sampled peatland, particularly during migration. In addition, *Cs. morsitans* has experimentally been demonstrated to be vector-competent for several *Plasmodium* species, including *P. circumflexum* and *P. vaughani* [8], which are among the *Plasmodium* species detected in this study. Therefore, *Cs. morsitans*/*fumipennis* might play a more important role in *Plasmodium* transmission than commonly thought.

In contrast, the *H. majoris* finding in *Cs. morsitans*/*fumipennis* with a recently ingested blood meal appears to be random, since it was only detected once in a single specimen (in this case, equivalent to one out of seven pools tested). In previous studies from Europe, *Haemoproteus* species were also detected in mosquitoes [32, 33]. However, investigations using extracts from whole mosquitoes—as in the present study—do not allow a conclusion on vector competence. Culicids are generally considered incapable of transmitting *Haemoproteus* parasites [1, 2], which was supported by Gutiérrez-López et al. [34], who experimentally demonstrated the lack of transmission by *Cx. pipiens* sensu lato. In addition, infections with *Haemoproteus* may even kill mosquitoes [35]. Therefore, although *Cs. morsitans* and *Cs. fumipennis* have never been tested for vector competence for *Haemoproteus* species, it is likely that the parasite was just ingested with the blood by the positive mosquito without being able to continue development. Unfortunately, it was not possible to determine the blood host of the positive sample.

All identified *Plasmodium* lineages have been previously documented in Europe, either within avian hosts or within mosquitoes, as catalogued in the grand lineage summary table of the MalAvi database (http://130.235.244.92/Malavi/). The most commonly detected were *P. relictum* SGS1, *P. matutinum* LINN1 and *P. vaughani* SYAT05, which at the same time belong to the most commonly detected *Plasmodium* species in Europe [15–19]. In Germany, *P. relictum* SGS1, *P. relictum* GRW 11, *P. circumflexum* TURDUS1 [12, 21], *P. matutinum* LINN1 [21] and *P. vaughani* SYAT05 [20, 21] have already been found in birds. The results of the present study now confirm the occurrence of these lineages in the German mosquito population as well. However, three distinct *Plasmodium* lineages (*P. cathemerium* PADOM02, *Plasmodium* sp. SYBOR02 and *Plasmodium* sp. PLOPRI01) have not been previously identified in Germany. Although each of these lineages was detected only once in the present study, their detection in field-collected mosquitoes is an indication of circulation and suggests a contribution to *Plasmodium* infections, even though their exact role in avian malaria remains unclear.

The MIR of each *Plasmodium* species varies from month to month, with the highest overall infection prevalence occurring in summer (July–August), representing the period with the highest infection risk for birds. This is in agreement with other studies from Europe. In Austria, the highest *Plasmodium* infection prevalence was also found in August [18]. Results from Switzerland suggest a higher chance of finding infected mosquito females in summer (July–August) than in spring (April–June) [16]. By contrast, the highest prevalence in Spain was found in autumn, probably due to a climate-related longer seasonal activity of mosquitoes as compared to Austria, Switzerland and Germany. In contrast to the studies from Austria, Switzerland and Spain, no *Plasmodium*-positive pools were found in Germany in April and May, which may be because of the small sample size during those months. The elevated MIR observed in September 2020 may again be attributed to the limited sample size. Overall, our study did not demonstrate a seasonal change in the prevalence of the various lineages as described by Lalubin et al. [16] and Schoener et al. [18].

Another interesting finding was the two *P. matutinum* LINN1-positive pools detected in November. The mosquitoes were collected in a hibernation shelter (cellar) in 2020 and 2021 at the same location. This result suggests that avian *Plasmodium* parasites may be able to overwinter in their mosquito vectors. This phenomenon is known for the human malaria parasite *P. vivax*, which can hibernate in *Anopheles* mosquitoes and infect humans during the winter season [36]. The overwintering of avian *Plasmodium* parasites could facilitate early-season transmission of the parasite when many birds breed, particularly affecting vulnerable nestlings. It may also explain *Plasmodium* transmission to juvenile birds during the breeding season [21]. According to an older study, it is unlikely for *Plasmodium* species, such as *P. relictum*, to survive the winter period within the mosquito vector due to temperatures below 12 °C that are considered lethal for the parasites [37]. Despite *Plasmodium* infections being known to have an adverse effect on mosquito fitness, primarily by reducing fecundity, it is still debated whether such infections positively affect mosquito longevity [38, 39]. The latter would certainly support parasite overwintering in mosquitoes, should temperatures be high enough for the parasites to survive. This finding definitely warrants further investigation, since it is known that different *Plasmodium* species are adapted to different temperatures, as, for example, human malaria parasites [40].

Based on the blood meal analysis, two different avian hosts were identified for two *Plasmodium*-positive specimens of *Cx. pipiens* biotype *pipiens*. These results imply that the mosquitoes were likely to have either ingested the plasmodia or infected their hosts with plasmodia while feeding on them. However, during the period of mosquito collection there were no signs of illness observed in either the black-headed ibis or the common eider populations of the respective bird parks (J. Westenfelder and G. Haase, pers. comm.).

The detection approach employed was not able to detect mixed infections. As double peaks occasionally occurred in the sequencing electropherograms, it is possible that some pools or single mosquitoes were positive for more than one *Plasmodium*/*Haemoproteus* species or lineage. Conversely, positive pools showing clear peaks in the electropherograms were apparently only infected with single *Plasmodium*/*Haemoproteus* lineages. This suggests that either a solitary infected mosquito was present in those pools, or multiple mosquitoes within the pools harboured the same parasite lineage. However, certain *Plasmodium* species may have also been underrepresented due to primer bias which can favour certain species over others in mixed samples [41]. Further studies using a combination of molecular and microscopic techniques are needed to investigate the prevalence of mixed infections and the impact of primer bias [41].

## Conclusions

The risk of avian malaria transmission is increasing with the rise in temperature caused by climate change, since ambient temperature has a direct effect on the reproduction and development of parasites within their ectothermic vectors [31]. The identification of potential vectors and the estimation of infection prevalence are crucial steps in the study of vector-borne diseases. Although the overall *Plasmodium* MIR found in this study (5.2) appears to be low, the ongoing circulation of *Plasmodium* parasites in the mosquito population of Germany was demonstrated. The study revealed the presence of eight distinct *Plasmodium* lineages, three of which (PADOM02, SYBOR02, PLOPRI01) have not been previously detected in Germany. This study also highlights the importance of considering potential natural avian *Plasmodium* vectors other than *Culex* species, such as *Cs. morsitans* or *Cs. fumipennis*.

## Data Availability

Data supporting the conclusions of this article are included within the article.
